# Simultaneous Remote Observations of Intense Reconnection Effects by DMSP and MMS Spacecraft During a Storm Time Substorm

**DOI:** 10.1002/2017JA024547

**Published:** 2017-11-03

**Authors:** A. Varsani, R. Nakamura, V. A. Sergeev, W. Baumjohann, C. J. Owen, A. A. Petrukovich, Z. Yao, T. K. M. Nakamura, M. V. Kubyshkina, T. Sotirelis, J. L. Burch, K. J. Genestreti, Z. Vörös, M. Andriopoulou, D. J. Gershman, L. A. Avanov, W. Magnes, C. T. Russell, F. Plaschke, Y. V. Khotyaintsev, B. L. Giles, V. N. Coffey, J. C. Dorelli, R. J. Strangeway, R. B. Torbert, P.‐A. Lindqvist, R. Ergun

**Affiliations:** ^1^ Space Research Institute Austrian Academy of Sciences Graz Austria; ^2^ Earth's Physics Department St. Petersburg State University St. Petersburg Russia; ^3^ Mullard Space Science Laboratory/UCL Dorking UK; ^4^ Space Research Institute RAS Moscow Russia; ^5^ Space Science Technologies and Astrophysics Research Institute Liege Belgium; ^6^ Applied Physics Laboratory The Johns Hopkins University Baltimore MA USA; ^7^ Southwest Research Institute San Antonio TX USA; ^8^ Institute of Physics University of Graz Graz Austria; ^9^ Heliophysics Science Division NASA Goddard Space Flight Center Greenbelt MD USA; ^10^ University of California Los Angeles, IGPP/EPSS Los Angeles CA USA; ^11^ IRF Swedish Institute of Space Physics Uppsala Sweden; ^12^ NASA Marshall Space Flight Center Huntsville AL USA; ^13^ University of New Hampshire Durham NH USA; ^14^ KTH Royal Institute of Technology Stockholm Sweden; ^15^ Laboratory for Atmospheric and Space Physics University of Colorado Boulder Boulder CO USA

**Keywords:** magnetic reconnection, plasma sheet boundary layer (PSBL), Magnetospheric Multiscale (MMS) mission

## Abstract

During a magnetic storm on 23 June 2015, several very intense substorms took place, with signatures observed by multiple spacecraft including DMSP and Magnetospheric Multiscale (MMS). At the time of interest, DMSP F18 crossed inbound through a poleward expanding auroral bulge boundary at 23.5 h magnetic local time (MLT), while MMS was located duskward of 22 h MLT during an inward crossing of the expanding plasma sheet boundary. The two spacecraft observed a consistent set of signatures as they simultaneously crossed the reconnection separatrix layer during this very intense reconnection event. These include (1) energy dispersion of the energetic ions and electrons traveling earthward, accompanied with high electron energies in the vicinity of the separatrix; (2) energy dispersion of polar rain electrons, with a high‐energy cutoff; and (3) intense inward convection of the magnetic field lines at the MMS location. The high temporal resolution measurements by MMS provide unprecedented observations of the outermost electron boundary layer. We discuss the relevance of the energy dispersion of the electrons, and their pitch angle distribution, to the spatial and temporal evolution of the boundary layer. The results indicate that the underlying magnetotail magnetic reconnection process was an intrinsically impulsive and the active X‐line was located relatively close to the Earth, approximately at 16–18 R_E_.

## Key Points

- Simultaneous observations of the PSBL during a strong substorm suggest a near‐tail reconnection site being located at 16–18 R_E_
- For the first time, we resolve both energy and pitch angle dispersion of the electron evidence that the boundary layer has temporal origin
- First observation of thin energetic electron layers in PSBL with thicknesses of the electron gyroscale

## 1 Introduction

Three decades ago, it was discovered (e.g., Eastman et al., 1984; Eastman, Frank, & Huang, 1985; Parks et al., 1984) that the outer boundary region of the magnetotail plasma sheet has specific properties, including complicated kinetic features which are different for electrons and ions, frequent appearance of unidirectional and bidirectional beams of both magnetospheric and ionospheric origins, and large‐scale and/or temporally variable structures. Many properties of this transition region lying between the plasma sheet and the lobe, called the plasma sheet boundary layer (PSBL), are well captured in the long‐term statistical survey covering many years, such as those carried out by Walsh et al. (2011) in which the regions were identified using Cluster observations (Escoubet, Fehringer, & Goldstein, 2001) ordered against the plasma beta parameter. The PSBL characteristics particularly include bidirectional electrons both in the lobe and outer plasma sheet, unidirectional earthward ion beam of high energy (tens of keV ions) at the outer plasma sheet (PS) edge which changes to a more bidirectional distribution deeper inside, before finally changing to the isotropic ion distributions characteristic of the central plasma sheet (CPS). It is worth mentioning that the characteristics of ions may vary case by case, as Grigorenko et al. (2009) reported that some PSBL crossings may not include ion beams.

**Figure 1:**
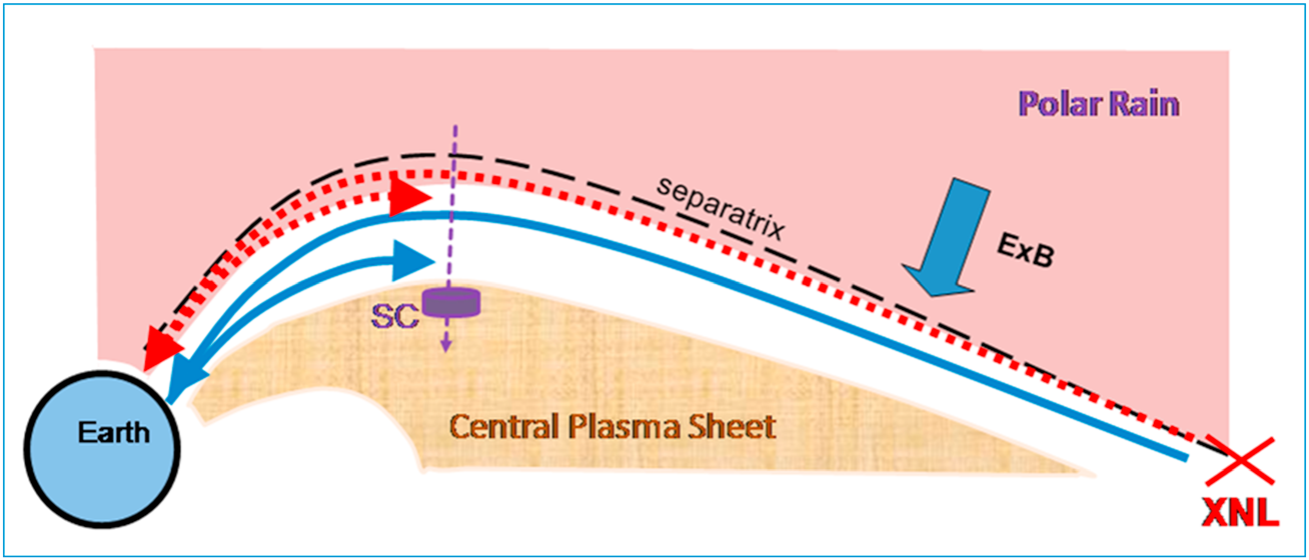
Sketch of particle beam motion under a steady magnetotail PSBL configuration and in presence of the reconnection‐induced convection. This illustration corresponds to a steady reconnection case, where the energy‐dispersed fast (slow) particles are shown by red (blue) colors (see section 1 for details).

It was reported by Takahashi and Hones (1988) that the appearance of unidirectional (earthward) energetic ion beams at the PSBL outer edge with an addition of a reflected (tailward) beam deeper in the plasma sheet can be understood using a simple reconnection‐based scenario. In this picture (see Figure 1), magnetic reconnection operating farther down the magnetotail drives the accelerated electron and proton beams, which move earthward along the magnetic field and spread along the magnetic field line according to their speed (the Time‐of‐Flight (ToF) dispersion effect) (e.g., Quinn & McIlwain, 1979; Sauvaud et al., 1999; Sauvaud et al., 2004; Sauvaud & Kovrazhkin, 2004). In near‐Earth space these particles are subsequently reflected back by the magnetic mirror effect and observed as a return beam. Simultaneously, the beam particles are convected perpendicular to the magnetic field lines towards the current sheet center plane at the *E* × *B* convection velocity, which provides the spatial dispersion of the particle beam known as the “convection velocity filter effect,” which provides a characteristic sharp low‐energy cutoff (e.g., Onsager et al., 1991; Sauvaud et al., 1999). The particle ToF flight time for particles in the incident beam is given by

(1.1) tToF=SVpar

where *S* is the distance between reconnection source and the observing spacecraft and *V*_par_ is the parallel component of the particle velocity. Computations assuming this simple scenario have demonstrated a quantitative agreement with observations as concerns the spatial ordering of proton beams, including the energy‐ and pitch‐angle‐depenent (e.g., direct and reflected beams) low‐energy cutoff behavior (Elphic et al., 1995; Onsager et al., 1991).

As for the electrons, there are indications that the electron edge of the PSBL lays lobeward of the ion edge and that their distribution also has a low‐energy cutoff, similar to the proton PSBL picture (Onsager et al., 1990). However, their temporal scale and spatial dispersion scale are much shorter. Consequently, the low‐energy cutoff pattern and its evolution could not be adequately resolved for the accelerated electron population by any previous mission before the Magnetospheric Multiscale (MMS) era. Thus, this is one of the goals of our present paper.

Besides predicting the spatial organization of accelerated energetic particles, the reconnection scenario also helps to identify and explain a sharp spatial boundary of low‐energy (few hundreds of eV) solar wind strahl electrons, which fill the open field lines nearly homogeneously, threading the magnetotail lobes and the polar caps (also known as polar rain (PR)) (e.g., Fairfield et al., 2008). As was first shown by Shirai et al. (1997), polar rain electrons passing near the X‐line are accelerated and shifted to a higher‐energy domain, therefore forming a sharp inner edge for particles of their original energy in space (see Figure 1). Once reconnection occurs, it cuts off the source of earthward traveling “nonaccelerated” portion of PR electrons. As a result of ToF, they may be observed with an energy‐dispersed upper energy cutoff. Thus, a flux gap forms at these energies on the spectrogram (between accelerated and lower energy part of polar rain population) in the vicinity of the separatrix; examples of which will be presented below in sections 2 and 3. Due to the convection, the inner edge is also velocity filtered, which produces the energy‐dispersed upper energy cutoff at locations inward from the separatrix (e.g., Alexeev et al., 2006).

The convection‐based velocity filter picture of Figure 1 is simple, and, if true, it can be used to extract from observational data some important characteristics of reconnection, for instance, its intensity and distance to the X‐line (Elphic et al., 1995), to provide a remote sensing of the reconnection process. In fact, the ion injection into the PSBL flux tube is often strongly modulated, as has become evident from observations by the Interball‐2 spacecraft (Sauvaud et al., 1999). At its ~2 *R_E_* apogee altitude, this high‐inclination spacecraft crossed the PSBL slowly, and during substorms it typically observed multiple sporadic yet recurrent injections of energetic (keV to few tens keV) magnetospheric ions. These injections have roughly a 1 min duration at a given energy with 1–3 min quasi‐period, if observed near the poleward border of auroral bulge. Such observations were interpreted as time‐of‐flight dispersed ion structures (TDISs) emitted by the equatorial source. A primary ToF origin was confirmed by their close association with the multiple activations of poleward expanding aurora and electrojets (Sergeev, Sauvaud, Popescu, Kovrazhkin, Liou, et al., 2000) as well as by comparisons with conjugate bursty bulk flow midtail observations (Sergeev, Sauvaud, Popescu, Kovrazhkin, Lutsenko, et al., 2000).

Another way to separate between true ToF and spatial velocity filter contributions to the energy dispersion is to compare the observations as a result of spacecraft motion during a PSBL crossing: during an inward crossing toward lower latitude (as shown in Figure 1) and an outward crossing toward higher latitude (opposite to that shown in Figure 1). For a pure ToF filtering, the energy dispersion should be always the same (high‐energy ions appear first), whereas for velocity‐filtered ions, as clear from Figure 1, the sense of energy dispersion is reversed for inward/outward crossings of the PSBL. The temporal and convection filter effects provide the same dispersion sense in case of inward crossing, but they have the opposite sense in the case of outward (poleward) crossing. Study of such outbound crossings at Interball‐2 apogees and Cluster perigees (~4 *R_E_*) complemented by multispacecraft comparisons showed that the transient ion injections are embedded within a broad region consistent with the latitude‐dependent convection‐filtered energy variation (Keiling et al., 2004; Sauvaud & Kovrazhkin, 2004). In addition, the multiple transient TDISs usually occupy a broad region of the plasma sheet, being observed at the expanding auroral bulge during substorms, with the impulsively activated plasma acceleration regions distributed along significant space of the midtail and distant plasma sheet. Note that the multiple localized acceleration regions are possible in the neutral sheet (Zelenyi et al., 2006). Hence, the entire picture of impulsive acceleration in the plasma sheet may be very complicated (Sharma et al., 2008, section 2.9).

In this paper, we concentrate on observations made by MMS in the PSBL region, where the well‐defined low‐energy proton cutoff features are observed at energies above several keV. The particular observations of interest here took place during a strong substorm event, when magnetic reconnection was active in the near‐Earth plasma sheet, at *r* < 20 *R_E_* (Nakamura et al., 2016). Using high‐resolution burst‐mode MMS observations at *r*~11.6 *R_E_*, we present the first observations of electron energy and pitch angle dispersion near the expected separatrix and consider its relationship to the proton dispersion. We also use plasma convection measurements, which were not available in previous studies of energy dispersion, and discuss the characteristics of magnetic field and electric field variations in relation to the dispersed electron and ion beams. A near‐simultaneous separatrix crossing by the DMSP F18 spacecraft provides observations of the energy dispersion of electrons and protons at low altitude and thus provides a more global context for our MMS‐based conclusions. These also allow a determination of the spatial dependence of the observed PSBL features, which is necessary for remote sensing of reconnection parameters. In the following sections, we start by discussing the large‐scale context and DMSP observations in section 2, then present detailed MMS observations in section 3 before discussing the implications of these in section 4. We summarize our conclusions in section 5.

## 2 Global Context and DMSP Observations

On 23 June 2015, a very strong substorm occurred around the time of the peak of a strong storm (*Dst*~−200 nT), with an expansion onset commencing at 03:16 UT. This period has been studied in detail by Nakamura et al. (2016), referred to hereafter as N16. The estimated total substorm current wedge (SCW) current exceeded 5 MA, resulting in a 1300 nT *AL* bay amplitude. This substorm included several substorm current wedge activations and step‐like expansions of activity (see Figure S1 in the supporting information and also Figure 1d of N16). Accordingly, in the premidnight sector of interest (22–23 magnetic local time (MLT)), the dipolarization observed at the geostationary GOES 13 spacecraft started soon after the onset (*B_h_* gradually increased from 03:17 UT; Figure 1f of N16), and an outward expanding plasma sheet was recorded after 03:27 UT (as sharp drop of the *B_y_* component and an energetic electron flux increase). At the separatrix crossing time, ~03:36 UT, the westward auroral electrojet at the 23 MLT showed both a large intensification and a poleward expansion up to ~70° geomagnetic latitude (see also Figure S1).

**Figure 2:**
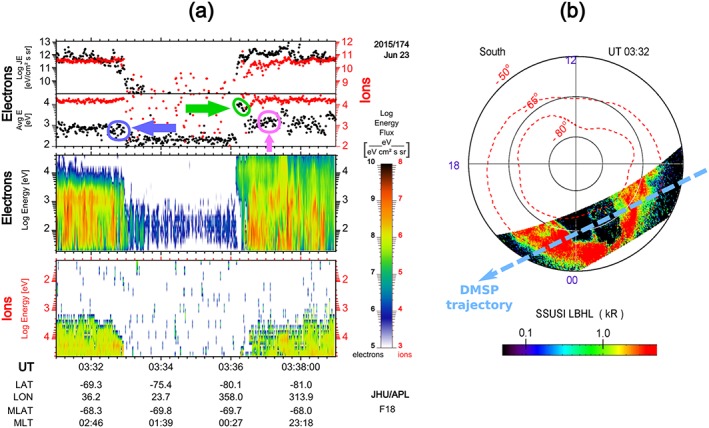
DMSP F18 observations during a storm time substorm on 23 June 2015. (a) The auroral particle observations from SSJ 5 instrument. (top to bottom) Differential energy flux of ions in red dots and electrons in black dots, average energy of ions and electrons, the energy spectrogram of electrons, and energy spectrogram of ions. Note that in the ion spectrogram the *y* direction is reversed so that ion energy is increasing down. The green arrow/oval on average energy plot (Figure 2a, second row) emphasizes the highly increased average electron energies on the plasma sheet entry at 03:36 UT, compared to the exit from PS at 03:30 UT marked by the blue arrow/oval. Also, the pink arrow/oval marks the lower energy equatorward portion of electrons. (b, right) The LBHL auroral image scanned across the DMSP F18 trajectory. The trajectory mapped to 120 km is shown approximately by the dashed blue line.

Moving along its trajectory from dawn into the premidnight sector in the southern hemisphere, the DMSP F18 spacecraft passed across the postmidnight auroral oval into the polar cap. It crossed the polar cap boundary (PCB) at 03:33 UT at about 01 MLT, which is shown in Figure 2. Intense fluxes of polar rain electrons with energy of a few hundred eV are seen throughout the polar cap segment of the F18 trajectory. The entry at ~03:36 UT from the polar cap into the oval is of primary interest in this paper. Two facts are important to note for this PCB crossing: first, the spacecraft crossed the morningside edge of the bright auroral bulge, which is strongly skewed toward dusk, being consistent with the SCW central meridian being between 21 and 22 MLT (see Figure 1d in paper N16). The poleward edge of the bulge is sharply defined both in the auroral emissions and in the precipitated electron energy flux. Second, the average electron energy at this PCB segment is roughly 10 times larger compared to that in the postmidnight plasma sheet (as emphasized by the green and blue arrows/ovals in Figure 2a). It is also larger than the electron energy in the equatorward part of the premidnight oval (marked with the pink arrow/oval on the plasma sheet electrons), which may indicate a strong acceleration process being in action near the separatrix at the time of PCB crossing. Note that the gap between the ion velocity filter and the polar rain transition is interpreted as a region of newly closed field lines on which the plasma sheet ions have not yet had the time to reach the ionosphere.

**Figure 3:**
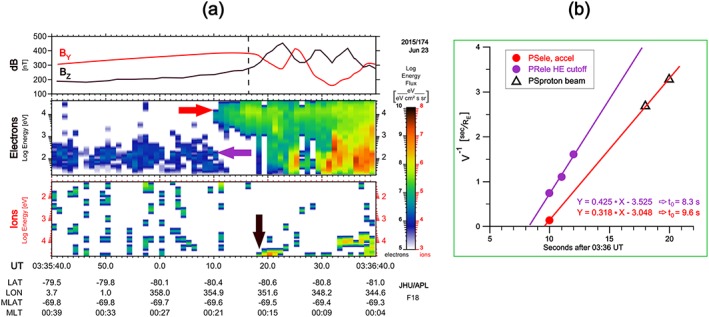
(a) High‐resolution view of particle spectra and magnetic perturbations at DMSP during the polar cap boundary crossing are shown. In Figure 3a, the first row represents the magnetic field components, and the bottom two are, respectively, electron and ion energy spectrograms similar to Figure 2. The horizontal *B_z_* (*B_y_*) components are measured across (along) the spacecraft trajectory correspondingly. The increasing *B_z_* indicates a field‐aligned current in the southern hemisphere. Three arrows in red, purple, and black, respectively, mark the observation of first PSBL electrons, cutoff in PR electrons, and arrival of dispersed proton. These three populations are chosen for the calculation of the Time‐of‐Flight (ToF) dispersion analyses. (b) The red, purple filled circles, and black triangle represent the same three populations and are plotted. The red and purple regression lines in Figure 3b are then used to calculate the injection time *t*_0_ and the distance from X‐line *S*_nl_ (see section 2 for details).

Figure 3a includes the particle spectra from the data collected by DMSP during this PCB crossing. From the details we will infer a few more distinct reconnection signatures. The first clear signature is a distinct dropout of polar rain electron fluxes, explained as being due to their sudden acceleration to high energies while crossing the separatrix near the X‐line (e.g., Alexeev et al., 2006; Onsager et al., 1990; Shirai et al., 1997). In agreement with that scenario, the dropout starts simultaneously with the first appearance of accelerated (~10 keV) electrons. The energy‐dispersed high‐energy polar rain electron cutoff timings (shown as purple points on Figure 3b) allow us to deduce the separatrix crossing time ~03:36:08.3 UT. The first appearance of the accelerated PSBL (~10 keV) electrons at 03:36:10 UT is close in time (within ~2 s) of the start of the energy‐dispersed polar rain dropout. The first appearance of accelerated proton beam occurs later, at 03:36:18 UT, but only in the two highest‐energy channels (30 and 20.4 keV). The red line in Figure 3b connects the first appearances of accelerated electron and proton beams, and its slope appeared to be very close to the slope of polar rain energy‐dispersed upper cutoff. This indicates that all three populations (i.e., accelerated electron and proton and disappearing polar rain) could be affected in almost the same flux tube, at the same time and at the same position, which is consistent with being a reconnection process. As concerns the observation of magnetic perturbations, they seem to start at 03:36:18 UT, nearly with the first appearance of high‐energy protons, and about 8 s after the first appearance of the electron beam. The fluctuation in the *B_z_* component of the magnetic field in Figure 3a indicates that the field‐aligned current (FAC) appears first during the PCB crossing and consistent with the bright auroral boundary in Figure 2b.

As mentioned in section 1, previous investigations suggested a temporal origin of energy‐dispersed proton beam variations observed near the outer edge of the poleward expanding auroral bulge (Keiling et al., 2004; Sauvaud & Kovrazhkin, 2004). These studies have also confirmed that the transient ion injections are embedded within a broader region due to the fact that the low‐energy cutoff can be latitude dependent and convection filtered. These convective displacements directed toward the plasma sheet are proportional to the ToF of a particle along the magnetic field line from their acceleration region (X‐line) to the observation point, and as such, the temporal effect (along the magnetic field) and the velocity filter effect (across the field) are proportional to each other. These two are summed up if the spacecraft moves inward, forming together the resulting energy dispersion.

**Figure 4:**
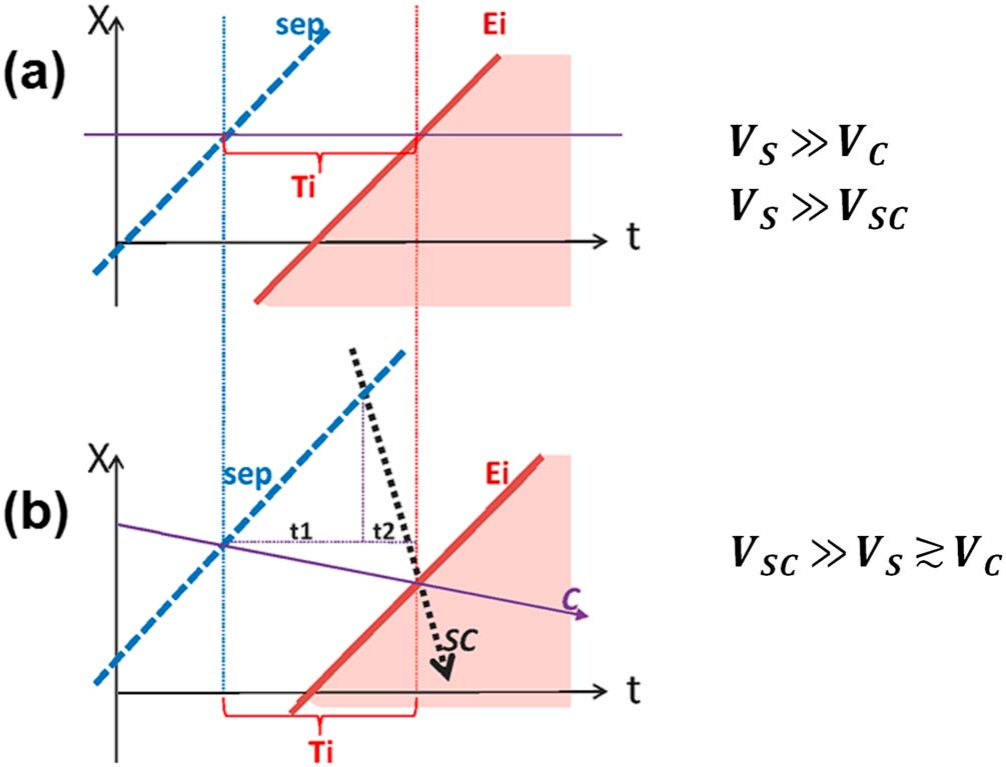
Sketch illustrating the kinematics of boundaries in case of (a) pure ToF and (b) the ToF combined with the convection. The **X** coordinate is perpendicular to both the magnetic field and separatrix surface; it lies in the surface passed through the spacecraft position. *Sep* denotes the separatrix mapped to that surface, and *E_i_* indicates the trajectory of low‐energy cutoff of specie *i* accelerated at separatrix; *C* indicates the convection trajectory, whereas *SC* indicates the spacecraft trajectory. Accordingly, *V_S_* is the motion of the separatrix, *V_C_* is the convection velocity of the plasma relative to the separatrix, and *V_SC_* is the velocity of the spacecraft. Here *T_i_* is the pure flight time calculated from equation (2.1), whereas apparent flight time *t*_2_ between separatrix crossing and particle cutoff crossings at the fast‐moving spacecraft given by equation (2.4) is much shorter (see section 2 for details).

Taking only the pure temporal affect into account (i.e., V^−1^ = *S*_nl_^−1^ × *t*_ToF_ from equation (1.1)), we use it to fit the onset times of electron/proton beams in Figure 3b. The fitted equation for the line of smaller slope is V^−1^ = 0.318 × (*T_i_* − 9.6), where *T_i_* is the pure flight time. This implies the apparent distance of these observations from the source to be *S*_nl_~3.1 *R_E_*, which is unrealistically short, placing the acceleration region into the strong dipole field. Note however that this is not a unique observation, as previously very short ToF distances of 2.4 *R_E_* was reported from DMSP observation during a strong near‐Earth reconnection event by Sergeev et al. (2007). In any case, we contend that the shortening of the apparent time delay originates from a combination of ToF and spatial effects, as presented in Figure 4 and explained below.

The diagram in Figure 4 shows the time evolution of nearly meridional 1‐D cut across the magnetic field near the ionosphere, where **X** is a meridional coordinate. In case of steady reconnection and no convection relative to the boundary, as shown in Figure 4a, the ionospheric projection of the separatrix (denoted as *Sep*) moves poleward at a constant velocity determined by the constant reconnection rate. The observer sitting at the fixed location (neglecting both the convection of plasma/field lines and the spacecraft motion) will monitor the precipitation coming from the same magnetic flux tube. After crossing the reconnection separatrix, the observer will start to see the particles accelerated by the reconnection after the time interval *T_i_*, corresponding to the particle travel time along the field line. Using equation (1.1), the pure ToF is given by

(2.1) Ti=SnlVi

where *V_i_* is the particle velocity of species *i* (either proton or electron of some specific energy) and *S*_nl_ is the distance from the reconnection line to the observation point, at the time of separatrix crossing.

Figure 4b illustrates the difference in boundary observation times, if we take into account the plasma convection velocity relative to the boundary (*V_C_*), as well as the fast inward motion of the observing spacecraft (at the velocity *V_SC_*, shown by dotted black line); which crosses the boundary from the polar cap into the auroral precipitation region. It shows that the beam particles registered by the spacecraft were again accelerated *T_i_* ago, but it is now longer (*t*_1_ + *t*_2_) compared to the apparent time delay *t*_2_ between the times, when the spacecraft first crossed the separatrix and subsequently encountered the particle beam. Taking into account that

(2.2) $$\Delta X_{S}=\Delta X_{\mathrm{SC}}-\Delta X_{C}$$

where

(2.3) $$\Delta X_{S}=V_{S}.t_{1},\Delta X_{\mathrm{SC}}=V_{\mathrm{SC}}.t_{2},\text{and}\Delta X_{C}=V_{C}.T_{i}$$

This gives an apparent time delay between separatrix and beam crossing as

(2.4) t2=Ti.VS+VCVS+VSC

In case of a low‐altitude spacecraft (DMSP‐like: 3.6°/min ~ 440 km/60 s), its meridional velocity is stable and relatively high at about *V_SC_* = 6 km·s^−1^, and thus, the convection and separatrix velocities typically have much smaller values. Taking the velocity of poleward auroral expansion ~1–1.5 km·s^−1^ under strong geomagnetic activities, it results in a 6–10° poleward expansion during a 10 min interval (e.g., Akasofu, 1976; de la Beaujardiere et al., 1994). Such poleward expansion of *V_S_* ~ 1 km·s^−1^ seems to be in agreement with the poleward expansion of westward electrojet observed between 03:28 and 03:34 UT at the meridian ~23 h MLT, which was about 1 h MLT westward of the DMSP F18 crossing (see Figure S1).

As for the equatorward convection flow and the corresponding equatorward drift speed of auroral arcs, on the nightside it is typically low and about a few tenths of km·s^−1^, and during substorm expansions the equatorward arc drift speeds in the poleward part of the auroral bulge are typically about *V_C_* = 0.4–0.5 km·s^−1^ (for both small and strong substorms) (see Kornilova et al., 1997; Figure 4). Under such condition where *V_C_* = 0.5 km·s^−1^ and *V_S_* = 1 km·s^−1^, the factor shortening the apparent travel time and distance would be

(2.5) t2Ti=1.57,≈0.21

In spite of the absence of actual measurements of poleward expansion and equatorward convection velocities, near the ionosphere the relationship *V_SC_* ≫ *V*_*S*_ ≳ *V*_*C*_ makes the above distance estimate to be rather conservative. Taking the above conditions into account, the actual distance of the acceleration point in the analyzed PCB crossing will be 3.1 × 5 ~ 16 *R_E_* from DMSP spacecraft, or in other words, the analysis here suggests the reconnection line to be at ~16 *R_E_* geocentric distance in the magnetotail.

## 3 MMS Observations

### 3.1 MMS Data and Position

We use Magnetospheric Multiscale (MMS) (Burch et al., 2015) observations on 23 June 2015, during the time when the spacecraft was located near the southern edge of the near‐Earth plasma sheet, at ~03:35 UT. During the interval of interest, MMS was located at [−10.3, 5.3, 0.4] *R_E_* GSM in a string‐of‐pearl configuration. This period of time was during the commissioning phase; hence, only certain instruments were actively collecting data, as also mentioned in N16 paper. For this study, the 3‐D particle data are obtained by the two fast plasma investigation (FPI) sensors: dual‐ion spectrometer (DIS) and dual‐electron spectrometer (DES) (Pollock et al., 2016). During the event of interest, the FPI was running on MMS2 and MMS3 under burst mode and returned ion and electron measurements, respectively, at 150 and 30 ms cadence. The fluxgate magnetometers (FGMs) (Russell et al., 2014) provided the DC magnetic field measurement at 16 Hz on all four spacecraft. The Spin‐Plane Double‐Probe (SDP) electric field instrument (Ergun et al., 2016; Lindqvist et al., 2014) delivered the electric field on MMS4 at 128 Hz and spacecraft potential on MMS3 at 32 Hz.

### 3.2 MMS Entry to the PSBL

As mentioned in section 2, the onset of the substorm commenced at 03:16 UT. MMS made a transient entry into the PSBL between 03:32:52 and 03:34:52 UT, based on which N16 did a detailed analysis on the transient and localized field‐aligned currents (FACs) within this time interval. Afterward, MMS temporarily returned to the lobe plasma until 03:35:14 UT, where the spacecraft made a final encounter with the PSBL. This latter PSBL crossing is the main focus of MMS observations in this paper.

**Figure 5:**
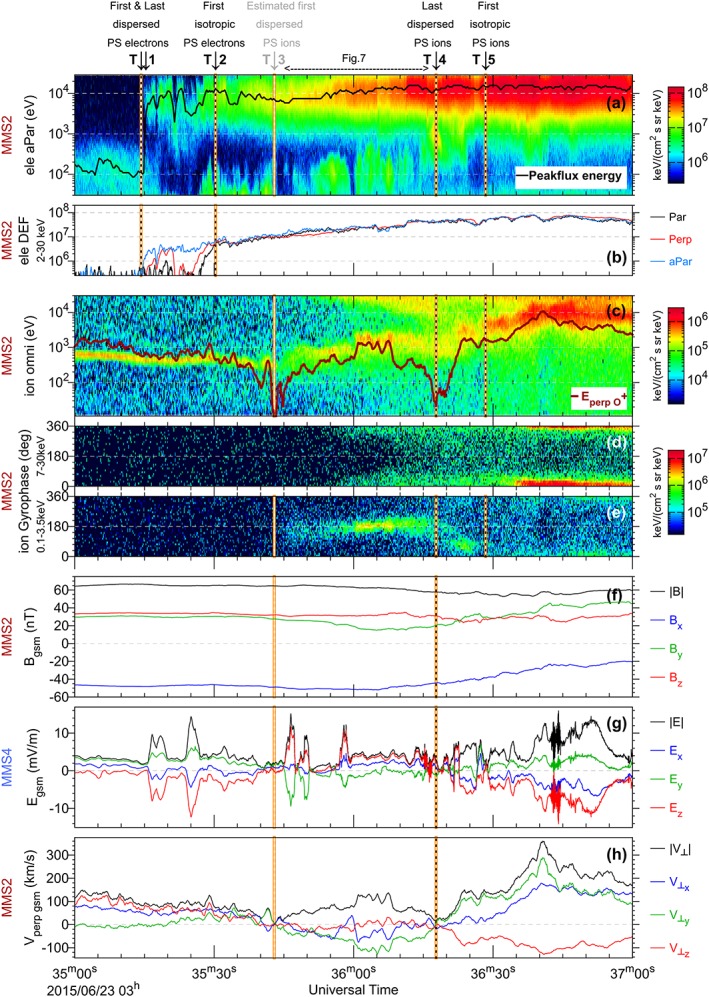
Overview of MMS observation of PSBL. (a) The energy spectrogram for antiparallel moving electrons and (b) differential energy flux for electron population between 2 and 30 keV. The parallel, perpendicular, and antiparallel populations are, respectively, in black, red, and blue line plots. (c) MMS2 omnidirectional energy spectrogram of ions, with overplotted red line indicating convecting O^+^ population. (d and e) The gyrophase of the perpendicular ion population. (f) The local magnetic field in GSM from MMS2 and (g) the local electric field in GSM, measured by MMS4. (h) The perpendicular velocity of ions measured by MMS2 (see section 3.2 for details).

Figure 5 represents an overview of plasma and field variations between 03:35 and 03:37 UT on 23 June 2015, as the spacecraft made an entry to the PSBL. In this figure, Figure 5a shows the energy spectrogram of antiparallel moving electrons from the MMS2 DES instrument for energies of the photoelectrons at 30 eV, with a pitch angle range of PA = 150°–180°. The black line which is overplotted on the spectrogram indicates the energy corresponding to the peak flux. Figure 5b represents the differential energy flux for three electron populations: parallel, perpendicular, and antiparallel with energies between 2 and 30 keV. They are calculated from pitch angle ranges of 0°–30°, 70°–110°, and 150°–180° from the MMS2 DES instrument and shown in black, red, and blue trace lines. Figure 5c shows the omnidirectional energy spectrogram for the ion population from the MMS2 DIS instrument. The overplotted red line shows the corresponding energy for O^+^ population, perpendicular to the magnetic field. Note that the FPI DIS instrument cannot distinguish between ion species. However, since both O^+^ and H^+^ are convected with *E* × *B* velocity, we can compare their energies in the spectrogram. *E*_perp_ calculated for O^+^ ( $E_{\text{perp}}=\frac{1}{2}\;m_{\text{oxygen}}.v_{⊥}^{2}$) matches with the spectrogram from FPI; hence, the population is expected to be predominantly O^+^. Figures 5d and 5e demonstrate the gyrophase (in field‐aligned coordinates) of the perpendicular ion population for high and low energies, respectively, 7–30 keV and 0.1–3.5 keV. The plots for low‐energy ions represent the direction of the convecting population, where the 0° indicates the flow directed toward ${\hat{b}}_{\mathrm{GSM}}\times {\hat{y}}_{\mathrm{GSM}}\times {\hat{b}}_{\mathrm{GSM}}$. Therefore, for this time interval, 0°/360° is predominantly directed toward dusk and 180° toward dawn. Figure 5f shows the local magnetic field in GSM, measured by the MMS2 FGM instrument, and Figure 5g shows the local electric field in GSM, measured by the MMS4 SDP instrument, and smoothed to magnetic field resolution. Finally, at the bottom, Figure 5h shows the convection velocity calculated from the ions moving perpendicular to the magnetic field, in the spacecraft frame, measured by the MMS2 FPI DIS instrument. An important caveat is that the ion velocities are determined by integrations over all species, assuming that they are predominantly protons H^+^. This potentially can lead to errors in the bulk velocities when there is a significant O^+^ population; however, when ions are moving in more or less the same direction, the direction that DIS reports for the bulk flow is trustable.

Between 03:35:00 and 03:35:14 UT, MMS2 observed a low‐energy electron population (<800 eV), accompanied by a narrow distribution of oxygen ions O^+^ (300–600 eV), while crossing the tailward magnetic field [*B_x_*, *B_y_*, *B_z_*] ~ [−48, 31, 34] nT. This is consistent with the spacecraft being in the southern lobe, where polar rain electrons are observed together with ionospheric outflows, with no plasma sheet population. At ~03:35:14 UT, which is labeled as T1 on the top of the figure and marked by orange dashed line on the plots, the polar rain population starts to disappear with a decreasing high‐energy cutoff, and simultaneously, the separate plasma sheet higher‐energy population (2–30 keV) appears showing a sharp low‐energy cutoff. As shown by the dominant fluxes of antiparallel electrons (blue trace line in Figure 5b), the dispersed population was traveling antiparallel, which was directed earthward. Note that the cutoff in cold polar rain lasts ~4 s, while for the higher‐energy plasma sheet population it was less than 2 s.

The label T2 at the top of the plot highlights the first time (~03:35:30 UT) when electrons from the plasma sheet show an isotropic distribution. In other words, same flux is observed for field‐aligned, perpendicular, and antifield‐aligned particles as indicated by the blue, red, and black lines in Figure 5b. It is worth mentioning that between T1 and T2, MMS2 observed several dropouts in the plasma sheet population, along with a brief emergence of a dominant perpendicular population. Throughout this interval, the magnetic field and the perpendicular velocity of ions remained almost unchanged, although electric field components, and in particular *E_z_*, increased twice between T1 and T2. Note that this electric field is measured at another spacecraft (MMS4) and is calculated from 2‐D measurements in ~ XY plane with the assumption that *E* · *B* = 0, meaning that the relevance of this *E_z_* measurement should be considered with caution. With these two caveats in mind, the peaks in electric field roughly line up with periods that the perpendicular electron population appears.

From T2 (~03:35:30 UT) onward, until T5 (~03:36:28 UT), the high‐energy plasma sheet electron population is continuously present and exhibits a monotonic increase in energy flux (from ~8 × 10^6^ to ~8 × 10^7^ keV/(cm^2^ s sr keV)) and in the energy of peak flux (from 10 to 15 keV). Throughout the whole interval, the low‐energy population (<1 keV) alters in flux and energy; however, we mainly focus on the high‐energy plasma sheet population. Note that observation of PSBL electron population with energies >30 keV has not been possible due to the upper energy limit for FPI DES instrument.

At around 03:35:43 UT, which is also labeled as T3, the duskward flow of ions reaches its first minimal value ~0 (Figure 5c). At this time the dawnward flow of low‐energy ions starts to appear (Figure 5e), while the magnetic field components start to show some perturbations (increase in *B_x_* and decrease in *B_y_*; Figure 5f), and *E_z_* and *V_y_* components change sign (Figures 5g and 5h). Note that these gyrophase plots only show the portion of flux that are nearly perpendicular to the magnetic field (PA = 70°–110°), and thus, when the parallel component of the particle velocity is significant, there is no flux shown. Prior to T3, the low‐energy ions had a velocity component along the magnetic field as well as perpendicular to the field and therefore not shown in gyrophase plots; however, after T3 the convection velocity of ions dominates over its parallel component. Between T3 (~03:35:43 UT) and T4 (~03:36:17 UT), the ion population shows a coexistence of low‐ and high‐energy ions (Figure 5c). In particular, at ~03:35:45 UT the dispersed high‐energy plasma sheet ions are observed with a low‐energy cutoff which lasts until T4. Within this period, the plasma sheet ion flux directed toward dusk started to appear at ~03:35:55 UT and remained peaked in the same direction until the end of the observations shown (Figure 5d).

T4 marks the observation of the last dispersed plasma sheet ions, which is accompanied by the second minimal value dawnward flow of ions ~0 (Figure 5c), and another sign change in *E_z_* and *V_y_* components (Figures 5g and 5h). It is worth mentioning that the electric field exhibits several variations in *E_z_* and *E_y_*, which may be related to low‐energy electrons and various possible current layers reported by N16. From T4, the low‐energy ions gradually merge into the higher‐energy population of the plasma sheet (Figure 5c), and their flow direction rotates gradually from dawn to dusk by T5 (Figure 5e). At T5, the plasma sheet ion population became isotropic and reached its maximum energy flux (Figures 5c and 5e). The fact that these changes in ion distribution occur after the dispersion stops at T4, together with the increase in *B_x_* between T4 and T5 suggests a spatial variation as MMS was moving toward the central current sheet. Note that the ion observations are also limited to FPI DIS upper energy channel at 30 keV, and the dispersed ions may exist beyond the energy range of DIS. After T5, the magnetic field components exhibit their maximum variations within the whole interval of the plot, in particular in *B_y_* components, and reach the values of ~ [−20, 45, 33] nT, by the end of the interval.

Overall, the observations in Figure 5 represent an overview of the MMS2 entry into the PSBL, moving from a location on the southern lobe open magnetic field before T1, to being on closed plasma sheet field lines after T5. Through this encounter, MMS2 observed the dispersion of beams of electron in the PSBL (T1 to T2) via a steady convecting magnetic field, and in the absence of PSBL ions. Then the spacecraft observed the dispersion of ion beams as it crossed the inner side of PSBL (T3 to T4), where the perturbation in the magnetic field became visible. Within this layer we see a mix of cold ions convecting toward dawn, and the high‐energy plasma sheet populations drifting toward dusk. Finally, the spacecraft settled inside an isotropic plasma sheet‐like ions after T5, where particles have already been bouncing between their magnetic mirror points, the flux and peak energy of electrons reached their maximum, and the cold oxygen ions from the lobe origin disappeared. We now present more details of the low‐energy cutoffs in ions and electrons observed by MMS within the PSBL layers.

### 3.3 Energy Dispersion at PSBL

During the observations of the PSBL by MMS, the spacecraft were close to apogee and therefore traveling at a relatively low speed of *V_SC_* ~1.5 km·s^−1^. Since MMS was operating in a string‐of‐pearl configuration, the four‐spacecraft timing analysis is not applicable for estimation of the boundary motion. Instead, we use minimum variance analysis on the magnetic field (MVAB) (e.g., Sonnerup & Cahill, 1967; Sonnerup & Scheible, 1998) to find the normal of the boundary and then use MMS2 and MMS3 separation to calculate the motion along the normal. The results of MVAB with constraint <*B*> ∙ *n* = 0 show that normal vector of the PSBL was *N* = (0.389, −0.334, 0.858) GSM. The delay time between of magnetic field variations measured by MMS2 and MMS3 between T1 and T5 was ~0.3 to ~0.5 s. By projecting the separation of two spacecraft ~ (−18.1, 87.2, −5.5) km in GSM onto the normal vector, we find the separation along the normal vector (~41 km), and then divide it by the delay time to estimate the normal motion of the PSBL: ~82 to 137 km·s^−1^. Assuming that the boundary was moving with the same speed as separatrix in the normal direction, then *V*_*S*_ ~ 82–137 km·s^−1^. This result is similar to previous studies that also show that an average PS expansion velocity of 130 km·s^−1^ was found during substorms (e.g., Forbes et al., 1981).

**Figure 6:**
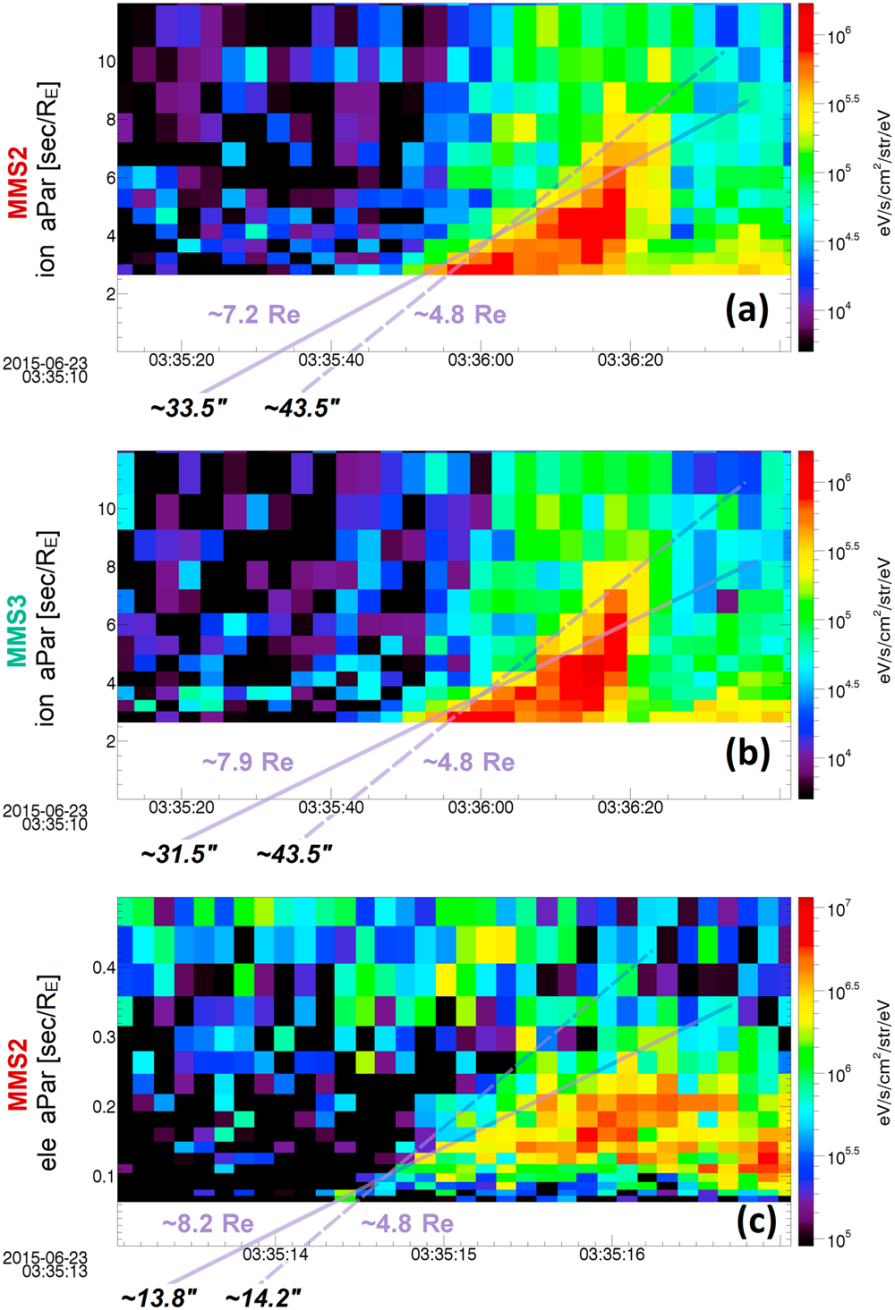
Energy dispersion of ions and electrons. (a and b) The energy flux of the antiparallel ions (earthward) measured at by MMS2 and MMS3, at 3 s temporal resolution. In these plots the *x* axis represents the time and *y* axis the reciprocal speed of the particle (V^−1^) in [s/*R_E_*] units. The two purple lines demonstrate the minimum and maximum slopes of the dispersion, respectively, as solid and dashed lines. (c) The first observation of dispersion of electrons from MMS2 at the temporal resolution of 120 ms. The estimated maximum distance revealed by ion dispersion and the injection times of ions are labels at the bottom end of the slopes (see sections 3.3 and 2 for details).

The convection velocity relative to the boundary can be calculated by subtracting the drift velocity, *V*_perp_, from the motion of the boundary *V*_*C*_ = *V*_*S*_ − *V*_perp_ . From Figure 5h, it is evident that *V*_perp_ was ~80–120 km·s^−1^ at the entry to PSBL and very similar to *V*_*S*_. As a result, the relative convection velocity at MMS position was much smaller than the velocity of separatrix, *V*_*S*_ ≫ *V*_*C*_. Also, the velocity of MMS was *V_SC_* ~1.5 km·s^−1^ and indeed much smaller than the velocity of separatrix, meaning *V*_*S*_ ≫ *V_SC_*. Considering *V*_*S*_ ≫ *V*_*C*_ and *V*_*S*_ ≫ *V_SC_*, equation (2.4) would give *t*_2_ ~ *T*_*i*_, resulting in an almost pure ToF effect. This means that we are able to apply the ToF calculations explained in section 2 and illustrated in Figure 4a to interpret the dispersion of electron and ion population observed by MMS. Figure 6 consists of three dispersion plots, where *x* axis is the time and *y* axis is the reciprocal speed V^−1^ of the particle in [s/*R_E_*] units. Figures 6a and 6b show the energy flux of the antiparallel ions (earthward beam), respectively, measured by FPI DIS instrument for MMS2 and MMS3. The data are originally collected at 150 ms temporal resolution within the PSBL. However, since the time interval of energy dispersion for ions is ~35 s, the data are averaged to 3 s temporal resolution. On both plots, two purple lines are drawn as estimations of the minimum and maximum slopes of the dispersion. The drawing of the slope lines are done by choosing pixels on the bottom left (first arriving dispersed particles) and top right (last arriving dispersed particles). The minimum line connects the two yellow pixels (>3 × 10^5^ keV/(cm^2^ s sr keV)) with the maximum distance between them, and the maximum line connects the minimum distance between two red pixels (>5 × 10^5^ keV/(cm^2^ s sr keV)). After the lines are drawn, an estimated distance from the injection point is determined labeled in purple, while the estimated injection times for the point the lines cross the *x* axis are written in black. The results from MMS2 and MMS3 agree on the minimum distance of ~4.8 *R_E_* with the injection time at 03:35:43.5 UT. The estimated maximum distances revealed by MMS2 and MMS3 are, respectively, ~7.2 *R_E_* and ~7.8 *R_E_*, and the injection times at 03:35:33.5 and 03:35:31.5 UT. Note that all spectrograms in this figure are plotted in energy flux units similar to previous studies (e.g., Keiling et al., 2004; Sauvaud et al., 1999), which may emphasize structures at higher energies versus using unites of phase space density. However, in this paper we do not focus on the minimum energy of dispersed population; rather, we only use the dispersion slope, which should remain unchanged regardless of the units.

The energy dispersion effect for ions has been extensively studied, as explained in depth in section 1. However, the dispersion of electrons has not previously been resolved by any spacecraft, as a high measurement cadence is required due to the high speed of the electrons. In our study, the 30 ms observations of electrons by the FPI DES instrument provide an unprecedented measurement for such observation. Figure 6c presents the dispersion of electrons from MMS2 at time interval which starts from the point marked as T1 in Figure 5, in similar format to those in Figures 6a and 6b. It is worth mentioning that the ion dispersion observation lasts ~35 s, while the time difference between the observation of the first and last dispersed electrons is less than 2 s. Here we average four consecutive measurements to have enough electrons counts at the temporal resolution of 120 ms. Similar to Figures 6a and 6b, two lines with minimum and maximum slope are drawn to calculate the distance from the injection point, and the corresponding injection time. Here the pixels are chosen by green (2 × 10^6^ keV/(cm^2^ s sr keV)) and yellow (3 × 10^6^ keV/(cm^2^ s sr keV)). The estimated minimum and maximum distances revealed from electron dispersion are, respectively, ~4.8 *R_E_* and ~8.2 *R_E_*, and their injection times are 03:35:13.8 and 03:35:14.2 UT.

The results of ions and electron put together suggest a distance of the spacecraft between ~4.8 *R_E_* and 8.2 *R_E_* from the injection point. It is worth mentioning that the similarity of the results for ion dispersion analysis between MMS2 and MMS3 suggests that both spacecraft observed the same ion beams. On the other hand, there is a considerable difference between the injection time of ions and electrons. In fact, the inferred injection time of the ions is a few seconds after the plasma sheet‐like electrons turn isotropic at T2, ~03:35:30 UT. Also, MMS2 was the only spacecraft which observed a clear dispersion in the electron observations, and MMS3 did not record sufficient electron counts for analysis during the first beam encountered, perhaps suggesting small‐scale differences in the fluxes in the PSBL electron layers. To understand this better, we investigate the similarities and differences between MMS2 and MMS3 electron observations in the following section.

### 3.4 Microscale Electron Structure in PSBL

**Figure 7:**
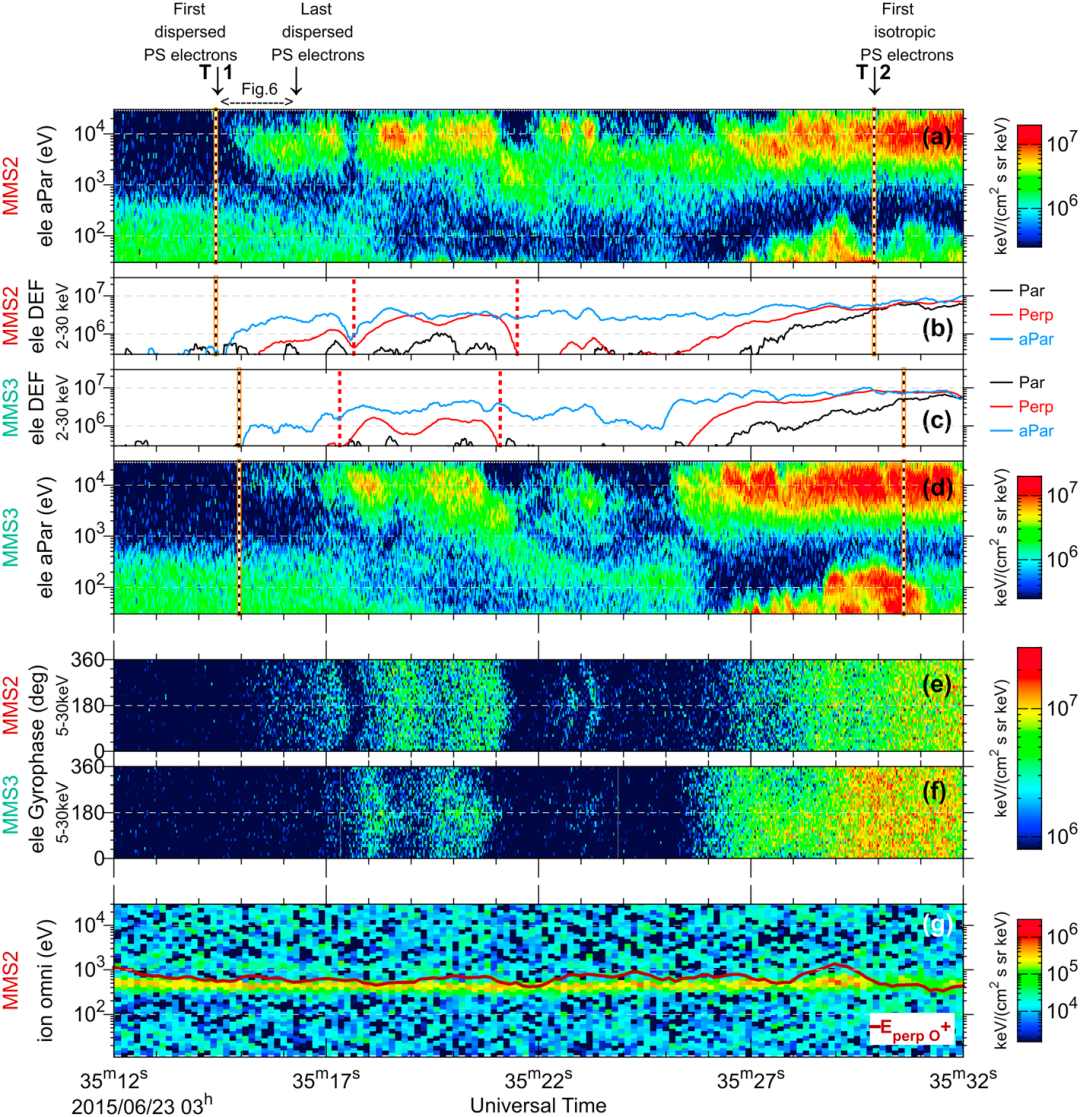
Comparison of electron beam observation by two MMS spacecraft. (a and d) Energy spectrograms for antiparallel moving electrons from MMS2 and MMS3 (energy level above the photoelectrons at 30 eV). (b and c) The differential energy flux for parallel, perpendicular, and antiparallel population with energies between 2 and 30 keV. (e and f) The gyrophase of the perpendicular high‐energy electrons (5–30 keV) for MMS2 and MMS3. (g) The MMS2 omnidirectional energy spectrogram of ions, with overplotted red line indicating convecting O^+^ population (see section 3.4 for details).

The observations of electron beams in PSBL (T1 to T2 region) by MMS2 and MMS3 are presented in Figure 7. In this figure, Figures 7a and 7d show the energy spectrogram for antiparallel (earthward) moving electrons from MMS2 and MMS3 (energy level above the photoelectrons at 30 eV), and Figures 7b and 7c demonstrate their differential energy fluxes for parallel, perpendicular, and antiparallel populations with energies between 2 and 30 keV. The times at which MMS2 observed the first PSBL electrons and the first isotropic population (respectively, T1 and T2 in Figure 5) are also marked by the vertical black and orange dashed lines in Figures 7a and 7b. MMS3 also observed the arrival of high‐energy PSBL electrons at ~03:35:15 UT. However, due to a low count rate the observation is patchy and the dispersion cannot be well resolved. MMS3 also observed the isotropic population toward the end of the interval (~1 s after MMS2), which is marked by vertical black and orange dashed line. Between T1 and T2, both spacecraft observed several variations in the flux of the electrons, dropouts in high‐ and low‐energy population, and variations in their temperature anisotropy and the energy of peak flux.

The similarities between MMS2 and MMS3 include (1) observation of polar rain prior to T1, emergence of the highest‐energy electrons seen in the PSBL after T1; (2) indication of a decrease in the high‐energy cutoff in the polar rain population after T1 until ~03:35:18 UT; (3) dropout in PSBL electrons around 03:35:22 UT and 03:35:25 UT; (4) appearance of low‐energy electrons at ~03:35:27 UT; and finally, (5) observation of the isotropic electron population with maximum flux at ~T2. We use the red trace lines in Figures 7b and 7c which represent the flux of the perpendicular population to compare the delay between the observation of similar electron layers. The two red vertical dashed lines in Figures 7b and 7c mark the leading and trailing edges of a layer of enhanced perpendicular flux (>10^6^ keV/(cm^2^ s sr keV)), which were observed between 03:35:17.66 and 03:35:21.50 UT by MMS2 and between 03:35:17.32 and 03:35:21.12 UT by MMS3. Here MMS3 was ~340 ms ahead of MMS2 in observation of the leading edge and ~380 ms ahead for the observation of the trailing edge. This systematic delay between the two spacecraft suggests a spatial variation, under a steady moving PSBL boundary (in agreement with observed ions in Figure 7g). Also, following the trailing edge of this layer, both spacecraft observed a similar midenergy population at ~1 keV with a dispersion slope.

There are also differences between the observations of the two spacecraft, which suggest that the electron layer contained microscale substructures. These differences include (1) the absence of a clear dispersion at T1 observation by MMS3; (2) dropout in the antiparallel population at ~03:35:17.30 UT, observed only by MMS2; (3) presence of the low‐energy polar rain‐like population between 03:35:21 and 03:35:25 UT, observed only by MMS3; and (4) the observations of different high‐energy populations between 03:35:21 and 03:35:26 UT, including a longer dropout for MMS3 versus shorter but multiple dropouts for MMS2. The relation of these differences to the spatial and temporal variations in the sources of the electrons will be discussed in the following section.

Similar to observations presented in Figure 5, the gyrophase of the perpendicular high‐energy electrons (5–30 keV) are shown in Figures 7e and 7f, respectively, for MMS2 and MMS3. The plots only show the electron population that have perpendicular fluxes higher than 10^6^ keV/(cm^2^ s sr keV) or in other words the intervals that the red trace lines in Figures 7b and 7c exceed 10^6^ keV/(cm^2^ s sr keV). The gyrophase of electrons prior to ~03:35:26/03:35:27 UT observed by MMS3/MMS2 show a characteristic bow‐shaped distribution “⦆,” which is absent afterward. Both spacecraft observed this feature at the leading and trailing edge of the layer indicated by red vertical dashed line (Figures 7b and 7c). In more detail, the observation of each layer starts/finishes with the bow‐shaped distribution, while the rest of the layers consist of a more gyrotropic distribution. The same feature also existed at ~03:35:17.25 UT, which was only observed by MMS2. The reason that such bow‐shaped distributions are observed is that, when spacecraft is at the entrance of a narrow flux layer, electrons with certain gyrophase which are all moving parallel to the boundary are the only flux being observed. But when the spacecraft is completely inside this flux layer, the electrons flux would be observed in all direction (gyrotropic), if the layer is thicker than few electron gyroradius. At the exit of the flux layer, electrons with gyrophase of 180° difference to those at the entrance are observed. This is an unprecedented observation of such within the electron layer of PSBL, resolved for the first time by high‐cadence FPI DES instrument on MMS.

MMS2 also observed two narrow bow‐shaped distributions at ~03:35:22.6 and ~03:35:23.2 UT, which seems to have been only partially observed by MMS3. These two narrow bow‐shaped distributions are particularly unique, as there is no gyrotropic population before or after them, which suggest an observation of a very narrow electron layer of only 1 gyroradius. This is equal to ~ (4–10) km and could not be resolved by the MMS2‐MMS3 separation at ~90 km.

Lastly, the bow‐shaped effects in the gyrophase plots (Figures 7e and 7f) are all in the same direction, as “⦆ ⦆ ⦆⦆”. This suggests that the flux layer was rather passing monotonically by MMS3 and then MMS2. Otherwise, if the MMS was moving back and forth the boundary, the bow‐shaped distribution would alter in direction, as “⦆ ⦅ ⦆⦆”. Note that the bow‐shaped distribution would have been only observable if the fluxes were forming a narrow flat layer. Otherwise, flux in a rather tube form would have resulted in a gyrotropic observation as seen toward time T2.

## 4 Discussion

During the poleward auroral expansion of a strong storm time substorm, DMSP and MMS spacecraft simultaneously crossed the expanding plasma sheet boundary and consistently recorded evidence of reconnection being in progress in the near‐tail region. Both spacecraft observed characteristics in ion and electron signatures as predicted for lobe/PSBL crossings earthward of an active lobe reconnection scenario, that is, the accelerated electron beams at the lobeward PSBL edge, the accelerated proton beam at a more inner location, and the energy‐dispersed high‐energy cutoff of the polar rain electrons. By analyzing quantitatively the variation of the energy‐dispersed low‐energy cutoff, a distance to the neutral line, *S*_nl_~16 *R_E_* was inferred from DMSP observations when taking into account a considerable shortening of apparent Time‐of‐Flight delay, as expected because of a large spacecraft velocity across the reconnection layers near the ionosphere, compared to both convection and separatrix velocities. Assumption of pure ToF origin of the dispersion provides similar results for the electron beam and for the proton beam observations at the MMS, suggesting a distance between MMS and X‐line of 4.8–8.4 *R_E_*; that is, the X‐line location is at *S*_nl_~16.4–20 *R_E_*.

An interesting observation of energetic population (~10 keV) of electrons near the separatrix provides one more argument in favor of very near‐Earth X‐line location. Assuming that there is no additional particle acceleration between the injection and observation point, the energy dispersion allows us to remotely predict the source region of the particle acceleration related to the local reconnection process. As known from theory and confirmed by both simulation and observational studies, (e.g., Haggerty et al., 2015), the energy gain of protons and electrons after passing the reconnection region is related to the lobe magnetic energy per proton‐electron pair as

(4.1) ΔE=ΔEproton+Eelectron≈0.3B022μ0n

where *B*_0_ is the magnitude of the reconnection field in the inflow region near the X‐line and *n* is the inflow plasma density at X‐line. For numerical estimates this gives

(4.2) ΔEkeV≈0.3B0nT/202ncm−3

Taking typical density values *n* ~ 0.1 cm^−3^ and supposing that the energy gain for protons and electrons is similar, and about 10 keV (clearly observed for electrons by both DMSP and MMS at the PSBL), one requires the lower estimate for the magnetic field in the lobe region near the X‐line to be *B*_0_ > 51 nT.

According to the adaptive modeling (Kubyshkina et al., 2011) results for this event (see Figure S2), such intense lobe magnetic field is only available at *S*_nl_ < 20 *R_E_* prior to the substorm onset, and at *S*_nl_ > 13 *R_E_* during the separatrix crossing time. Note that the model estimate is approximate since there was no spacecraft observations at >11 *R_E_* to constrain confidently the adaptive model in the tail region. Nevertheless, such estimate shows independently that strong electron acceleration can be achieved only if the reconnection operates in the near‐Earth magnetotail, where the large magnetic energy per particle in the inflow region is available.

The 30 ms electron observation by MMS facilitates a unique opportunity to examine the time sequence of electron observations with various pitch angles. In particular, we are interested in the time difference between the arrival of antiparallel electrons (PA = 180°) and the electrons with bouncing points near the position of MMS (PA = 90°). We take the two inferred X‐line distances from MMS2 (*S*_nl_ = 16.4 and 20 *R_E_*) and trace the electrons from the injection point to the observation point at MMS2. Here the assumption is that the conservation of the first adiabatic invariant is valid, and the reconnection occurs as a single X‐line scenario. Our numerical calculation shows that the delay between the arrivals of electrons ~20 keV with PA = 180° and PA = 90°, from the injection point with *B*_0_~50 nT, are, respectively, ~0.88 and ~1.15 s. The actual time delay between the arrival of PA = 180° and PA = 90° measured by MMS2 during the energy‐dispersed interval is 0.8–1.0 s (Figure 7b; the delay between the flux enhancement in blue and red trace lines at ~03:35:14.4 and ~03:35:15.4 UT). The consistency between the numerical calculation (particle tracing) and MMS2 observation suggests that the adaptive model has provided a realistic estimation for the background magnetic field at the reconnection site.

Note that pitch angle dispersion can also be possible due to the convection of inner layers of PSBL. Therefore, although we might not be able to confirm that both particles with PA = 180° and PA = 90° are from the same source, using the particle tracing calculation, we can confidently assume that electrons with PA = 90° from the same source could not have arrived sooner than Δ*τ*_1_ = 0.8–1.0 s after the arrival of electrons with PA = 180°. In a similar approach, we can also quantitatively compare the dispersion of pitch angle for the higher‐energy population versus the lower energy population of PSBL. MMS2 observations show that the electrons of ~2 keV with PA = 180° arrive Δ*τ*_2_~0.2–0.3 s earlier than ~20 keV with PA = 90°. Taking these two time delays Δ*τ*_1_ and Δ*τ*_2_ into account, the farthest possible X‐line has to be located at 18 *R_E_*, and the reconnecting lobe magnetic field *B*_0_ > 48 nT. The inferred X‐line position at *S*_nl_~16–18 *R_E_* is quite realistic for active conditions according to previous Geotail and Cluster surveys (Nagai, Shinohara, & Zenitani, 2015; Petrukovich et al., 2009). It is worth mentioning that a combined BATSRUS + RCM simulation successfully captured this substorm event and suggested that reconnection operated at distances <20 *R_E_* during a 03:20 UT substorm (see Reiff et al., 2016, Figure 5).

With a near‐Earth reconnection in the magnetotail being suggested from DMSP and MMS observations, we now can focus on the details of the PSBL layers observed by MMS. As seen in Figures 3a and 5f and explained in sections 2 and 3.2, both DMSP and MMS observed the perturbations in the magnetic field components as the first ion/proton population arrived. This is clearer in MMS that the field remains almost unchanged from T1 to T2, where electron layers are crossed, and it is only after T3 when then the ions arrive and the perturbations in the magnetic field components appear. If we compare the magnetic field (Figure 5f) with the perpendicular velocity (Figure 5h), there is a clear correlation between each components of *x*, *y*, and *z* as they decrease/increase at T3 and T4. This is consistent with previous reports ( e.g., Hirahara et al., 1998; Sotirelis et al., 1999; Takada, Seki, Hirahara, Fujimoto, et al., 2005; Takada, Seki, Hirahara, Terasawa, et al., 2005) that the magnetic perturbations start to be seen in between registered electron and ion beams and closer to ions. In particular, Sotirelis et al. (1999) also showed that a convection discontinuity appears far from polar rain boundary but near the outermost ion beam. In this case, IBL is the region between T3 and T4 that is observed almost identically by both MMS2 and MMS3.

Within the IBL, dawnward flow of cold ions and the duskward flow of high plasma sheet ions are observed, which is related to the vertical gradient of plasma sheet ion flux and finite‐gyro effect (e.g., Andrews, Keppler, & Daly, 1981; Daly & Keppler, 1983; Oksavik et al., 2002; Owen et al., 1995). Its stability indicates a stable orientation of the plasma sheet boundary surface during the interval of interest.

The minimum and maximum injection times in Figure 6 are calculated from the energy dispersion of earthward moving ions and electrons, antiparallel to the magnetic field. Considering the two pixel size approximation in the calculations, then for ions the injection time has a ±6 s error, and for the electrons ±0.24 s error. With that caveat in mind, these extrapolated injection times can provide two important pieces of information:

1. Based on the variation in the observed magnetic field, and also the first minimal at the perpendicular ion velocity, the first dispersed ions are estimated to have arrived at 03:35:42.5 UT (marked as T3 on Figure 5). This time is strongly in agreement with the injection time from the ToF analysis, for the closer X‐line at 03:35:43.5 ± 6 UT.
2. The injection of ions along the field seems to have taken place considerably later (~13–36 s) than those for the electrons. In fact, the electrons are already isotropic before then. We postulate that this is an evidence that the ions have been accelerated further away from that electron acceleration point at the reconnection site (perhaps several *d_i_*), hence the difference in injection times. We suggest that the sequence of the processes follows as (i) electrons are rapidly accelerated and leave the reconnection site; (ii) the X‐line typically moves toward the tail with the speed of ~0.1 outflow speed (e.g., Alexandrova et al., 2015) during which the ions could still be in the processes of acceleration; and (iii) ions reach their maximum energy, leave the reconnection site earthward, and their ToF suggest similar location for the X‐line but with time delay. However, it is not possible to estimate the distance from current remote observations in this paper. Future observations and or simulations could contribute to estimating the typical distance.

As mentioned in section 1, the inner edge of the PSBL is occupied by the ion boundary layer (IBL), as consistently evidenced by both DMSP and MMS observations. At the outer edge of the PSBL lies the electron boundary layer (EBL), which is the most relevant to the observations of electron structures presented in 3.4. The separation between MMS3 and MMS2~(−18.1, 87.2, −5.5) km in GSM is small enough that both spacecraft observed the same ion structure and very similar magnetic field (not shown), yet it has resulted in different observations in the details of electron boundary layer. As shown in Figures 7a and 7d, only MMS2 observed the dispersion of energies in electrons. This is either due to the fact that DES instrument on MMS3 has not had the chance to collect enough counts and or due to spatial variations in the electron flux in the *y* direction.

Observations in the EBL show an emergence of an earthward PSBL electron beam (low‐energy cutoff) simultaneously with the disappearance of the earthward polar rain electrons with a high‐energy cutoff. As the spacecraft enters inside the EBL, the tailward polar rain (i.e., mirrored population) also disappears. Here lays the FAC layer and transient field disturbance, which has been reported in past to be related to Hall effect of diffusion region (e.g., Fujimoto et al., 2001; Nakamura et al., 2004). To address the systematics of field‐aligned electrons at the lobe/plasma sheet boundary, Manapat et al. (2006) did a survey on WIND spacecraft data in the distant tail and showed that low‐energy electrons were directed toward and the higher‐energy electrons directed away from the X‐line. As a result they concluded that the Hall effect can be detected even at large distances from the diffusion region. We agree that the electron energy/PA pattern near the separatrix (or near PSBL) may be explained as a part of Hall current/convection system if observed very near the X‐line. However, (1) here the MMS and DMSP observations were made very far away from the X‐line and (2) consistent energy dispersions of proton and electron beams were observed indicating their common source and origin (proton beam dispersion has nothing common with Hall effect). In fact, the counterstreaming high‐ and low‐energy electrons of Manapat et al. (2006) can be explained as simultaneous registration on newly reconnecting field lines of reflected (tailward moving) low‐energy PR electrons and of earthward moving accelerated (high‐energy) PSBL electrons, with both experiencing a velocity filter effect.

Inside EBL, one notable structure is the period highlighted by red vertical dashed line (Figures 7b and 7c), which is observed by both MMS2 and 3. Since both the leading and trailing edges of this structure show same time delay, this seems to be a spatial structure in the EBL. The appearance of the electrons with PA = 90° (Figures 7b and 7c) is also consistent with a high‐flux layer of electrons passing by MMS, which have had enough time to bounce back from the magnetic mirror (southern hemisphere). Note that the absence of parallel electrons suggests that those electrons have not yet mirrored back from locations far from the observation site. The electric field activity (Figure 5g) also increases during the passage of this flux layer, which is perhaps due to the Hall effect, as the ion convection is steady and not affected by these electron layers.

Following the trailing edge of this narrow layer, both spacecraft observed a similar mid‐energy population at ~1 keV with a dispersion slope. This might be due to the fact that the dispersed electrons with the highest energies have passed the observation point prior to the arrival of MMS, but the lower energy populations are still observed, e.g., due to temporal flux variation.

Finally, we focus on the observation of electron flux layer with the thickness of only ~1 gyroradius by MMS2 and 3 at ~03:35:22.6 and ~03:35:23.2 UT. Two bow‐shaped features ⦆ are clearly observed by MMS2, while MMS3 only observed it partially. Before and after the time of observation of this layer, the earthward beams of electrons were also observed by both MMS2 and MMS3. However, the time delay of observation does not match with the other spatial structure we discussed earlier. In addition to that, during this interval only MMS3 observed the polar rain electrons simultaneously with PSBL electrons. Taking the steady convection (Figure 7g) into account, a possible explanation is that the difference of observations between that MMS2 and MMS3 is due to a temporal variation in the reconnection process at the X‐line. Under such scenario, MMS2 observed the electrons from one source, while MMS3 was on a more recently reconnected field line where polar rain electrons were still arriving from a second X‐line.

**Figure 8:**
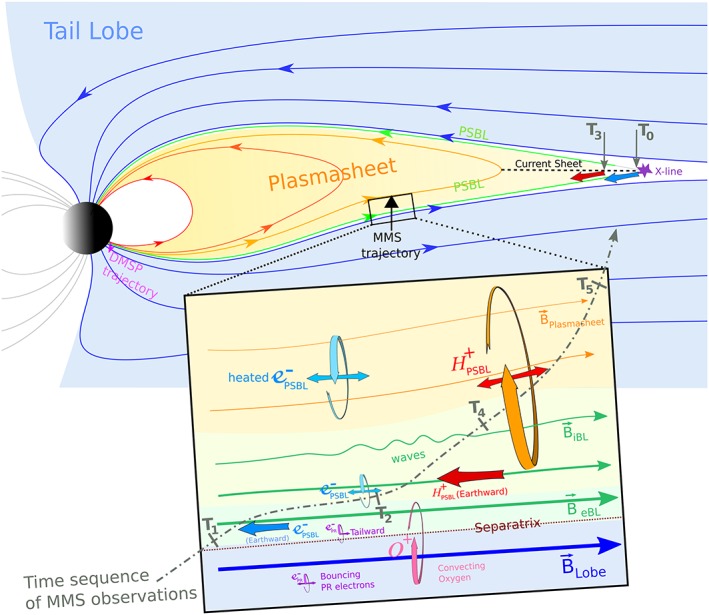
The sketch of the magnetotail and PSBL layering during 23 June 2015 storm. The trajectory of DMSP and MMS are indicated, respectively, in pink near the Earth and black at midtail. The bottom rectangle is a zoomed‐in view of what PSBL looked like when MMS crossed the boundary. The convecting oxygen O^+^ (pink) along with the polar rain electrons (purple) are present in the lobe region. At the outer edge of electron boundary layer (EBL), the strong PSBL earthward electron beams (dark blue) are observed, while the polar rain starts to disappear. In the inner edge of EBL the bouncing PSBL electron (light blue) heat up, hence the bigger gyroradius. At the ion boundary layer (IBL), the first beams of earthward ions (red) arrive and magnetic perturbations are being observed (green waves). The bouncing plasmasheet ions (orange) can be present at the IBL, as they have had enough time to convect inward of PSBL and earthward. The time sequence of the observations is shown by a dot‐dashed line and labeled as T1 to T5 from the observation (see Figure 5). Also near the X‐line, T0, which is 30 s sooner than T3, shows the time delay between electron injection time and ion injection time (see section 4 for details).

To summarize these observations, a sketch of the magnetotail PSBL is presented in Figure 8. In this sketch, the trajectories of DMSP and MMS are indicated, respectively, in pink near the Earth and black at midtail. The bottom rectangular sketch is a zoomed‐in view of how the PSBL was structured when MMS crossed the boundary. In this sketch, the magnetic field in the lobe, PSBL including EBL and IBL, and also plasma sheet are shown as tailward arrows. The convecting oxygen O^+^ (pink), along with the polar rain electrons (purple), are present in the lobe region, and they can enter the EBL based on their gyroradius scale. At the outer edge of EBL, the strong PSBL earthward electron beams (dark blue) are observed, while the polar rain begins to disappear. Inside the EBL, the electrons which have enough time to mirror and bounce back are observed, and at the inner edge of EBL the bouncing PSBL electron (light blue) heat up, hence the bigger gyroradius. Inside the IBL, the first beams of earthward ions (red) arrive and magnetic perturbations are being observed (green waves). The bouncing plasma sheet ions (orange) can be present at the IBL, as they have had enough time to convect inward of the PSBL and earthward. An important message is that these layers are not static, and both spatial and temporal variations affect the observation. Thus, we have overplotted the time sequence of the observations with a dot‐dashed line. The time sequence highlights that the highest temporal variation is observed with the EBL, while IBL was steadier during the time of the crossing.

## 5 Conclusions

1. During the poleward auroral expansion of strong substorm, the DMSP and MMS spacecraft simultaneously crossed the expanding plasma sheet boundary. The observations provide strong evidence that the reconnection was in progress in the near‐tail region at *r*~16–18 *R_E_*.
2. For the first time we were able to resolve the energy and pitch angle dispersion of outermost accelerated electron boundary layer and show evidence that energy dispersion in this layer has predominantly temporal origin.
3. For the first time we observed very thin layers of intense accelerated electrons in PSBL region, which are of as thin as ~1 electron gyroradius scale.
4. In this outermost electron boundary layer, there is of evidence spatial and temporal variations that suggest a second‐scale variations in the reconnection process. Together with previous knowledge of minute‐scale flux variations of accelerated protons, this indicates that magnetotail magnetic reconnection is an intrinsically impulsive process.
5. Different injection locations/times of electrons and ions is a strong indicator that the acceleration of ions take places away from acceleration point of the electrons at the reconnection site.

Such high‐resolution observations are crucial to understand the microscale physics of the PSBL. Future MMS observation, in particularly during the phase 2b and under a tetrahedral configuration, could contribute to further understanding of the spatial and temporal structures in the EBL.

## Erratum

In the originally published version of this article, the name of author T. Sotirelis appeared incorrectly in the author list as “T. Sotireli”. The error has been corrected, and the present version may be considered the authoritative version of record.

## Supporting information



Supporting Information S1
