# Supplementary material for: Simultaneous Remote Observations of Intense Reconnection Effects by DMSP and MMS Spacecraft During a Storm Time Substorm
Source: J Geophys Res Space Phys. 2017 Nov 3;122(11):10891–909. doi: 10.1002/2017JA024547 (PMC5784414; doi:10.1002/2017JA024547)
Supplement: Supplementary file 1 — Supporting Information S1 [file JGRA-122-10891-s001.docx]

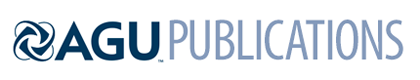


Journal of Geophysical Research - Space Physics

Supporting Information for

Simultaneous Remote Observations of Intense Reconnection Effects by DMSP and MMS Spacecraft during a Storm-time Substorm

A. Varsani^1^, R. Nakamura^1^, V. A. Sergeev^2^, W. Baumjohann^1^, C. J. Owen^3^, A. A. Petrukovich^4^, Z. Yao^5^, T. K. M. Nakamura^1^, M. V. Kubyshkina^2^, T. Sotireli^6^, J. L. Burch^7^, K. J. Genestreti^1^, Z. Vörös^1,15^, M. Andriopoulou^1^, D. J. Gershman^8^, L. A. Avanov^8^, W. Magnes^1^, C. T. Russell^9^, F. Plaschke^1^, Y. V. Khotyaintsev^10^, B. L. Giles^8^, V. N. Coffey^11^, J. Dorelli^8^, R. J. Strangeway^9^, R. B. Torbert^7,12^, P-A. Lindqvist^13^, and R. Ergun^14^

^1^ Space Research Institute, Austrian Academy of Sciences, Graz, Austria.

^2^Earth’s Physics Department, St. Petersburg State University, St Petersburg, Russia.

^3^Mullard Space Science Laboratory/UCL, Dorking, UK.

^4^Space Research Institute RAS, Moscow, Russia.

^5^Space science, Technologies and Astrophysics Research (STAR) Institute, Liège, Belgium.

^6^Johns Hopkins University, Applied Physics Laboratory, Maryland, USA.

^7^Southwest Research Institute, San Antonio, Texas, USA.

^8^NASA Goddard Space Flight Center, Heliophysics Science Division, Greenbelt, MD, USA.

^9^University of California Los Angeles, IGPP/EPSS, Los Angeles, CA, USA.

^10^IRF Swedish Institute of Space Physics Uppsala, Uppsala, Sweden.

^11^NASA Marshall Space Flight Center, Huntsville, AL, USA.

^12^University of New Hampshire Main Campus, Durham, NH, USA.

^13^KTH Royal Institute of Technology, Stockholm, Sweden

^14^University of Colorado, Laboratory for Atmospheric and Space Physics, Boulder, CO, United States

^15^Institute of Physics, University of Graz, Graz, Austria

**Contents of this file**

Figures S1 and S2

**Introduction**

The following figures which are mentioned in Section 2 of the paper, provide detailed magnetograms data, and well as the results of adaptive magnetospheric modeling during the separatrix crossing.


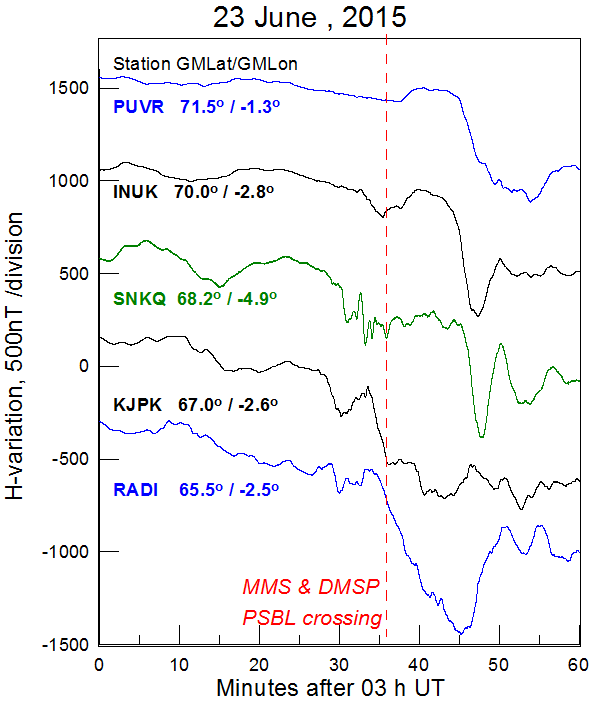


Figure S1. H-component magnetograms of meridional station chain located near ~22 h MLT meridian during the separatrix crossing time. After 03:30 UT magnetograms show a poleward progression of westward electrojet from ~65-67^o^ to 70^o^ GMLat as well as its strong intensification.


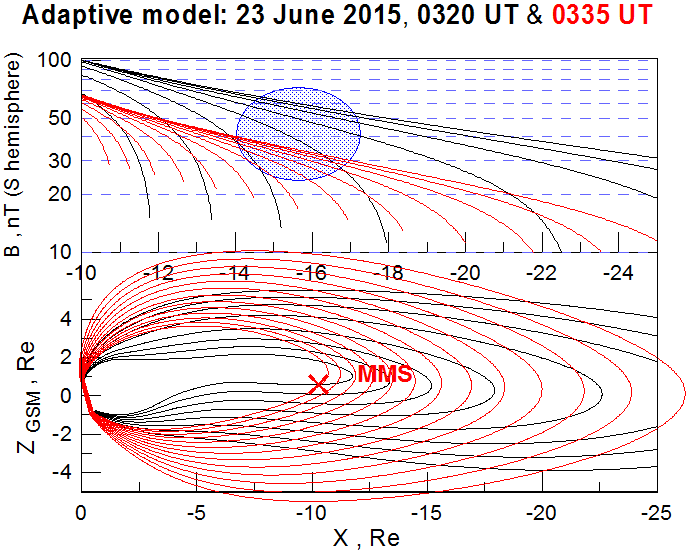


Figure S2. Results of adaptive magnetospheric modeling [Kubyshkina et al., 2011] at the start of substorm (03:20 UT, black lines) and during the separatrix crossing. The model was adapted to the actual measurements of GOES 13, GOES 15 and MMS spacecraft. Bottom panel shows magnetic field line configurations and MMS location. Top panel shows the distribution of magnetic field magnitude along the field lines which are plotted in the bottom panel (shown only for the southern hemisphere). Hatched region indicates the distance range of X-line locations as inferred from ToF analyses of electron and proton beam dispersion. The lobe magnetic field magnitude in this area is evaluated to be 50-65 nT prior to substorm expansion, and 30-40 nT during the separatrix crossing time.
